# RETRACTION: Manganese‐12 Acetate Suppresses the Migration, Invasion, and Epithelial–Mesenchymal Transition by Inhibiting Wnt/β‐Catenin and PI3K/AKT Signaling Pathways in Breast Cancer Cells

**DOI:** 10.1111/1759-7714.70015

**Published:** 2025-02-18

## Abstract

**RETRACTION:** H. Ju, Y. Li, X. Xing, X. Miao, Y. Feng, Y. Ren, J. Qin, D. Liu, Z. Chen, and Z. Yang, “Manganese‐12 Acetate Suppresses the Migration, Invasion, and Epithelial–Mesenchymal Transition by Inhibiting Wnt/β‐Catenin and PI3K/AKT Signaling Pathways in Breast Cancer Cells,” *Thoracic Cancer* 9, no. 3 (2018): 353–359, https://doi.org/10.1111/1759‐7714.12584.

The above article, published online on 08 January 2018 in Wiley Online Library (wileyonlinelibrary.com), has been retracted by agreement between the authors; the journal Editor‐in‐Chief, Tateaki Naito; and John Wiley & Sons Australia, Ltd. The retraction has been agreed upon following an investigation into concerns raised by a third party which revealed inappropriate image panel duplications between this (Figure 1a and b) and three other articles from the same institute published in a different scientific context. The authors admitted to the image compilation error and stated that during the experimental work, the author groups shared a laboratory which could have led to the error in reusing the images. Due to the number of errors identified in the published figures, the authors and the editors have lost confidence in the presented data and consider the conclusions substantially compromised.

**RETRACTION:** H. Ju, Y. Li, X. Xing, X. Miao, Y. Feng, Y. Ren, J. Qin, D. Liu, Z. Chen, and Z. Yang, “Manganese‐12 Acetate Suppresses the Migration, Invasion, and Epithelial–Mesenchymal Transition by Inhibiting Wnt/β‐Catenin and PI3K/AKT Signaling Pathways in Breast Cancer Cells,” *Thoracic Cancer* 9, no. 3 (2018): 353–359, https://doi.org/10.1111/1759‐7714.12584.

The above article, published online on 08 January 2018 in Wiley Online Library (wileyonlinelibrary.com), has been retracted by agreement between the authors; the journal Editor‐in‐Chief, Tateaki Naito; and John Wiley & Sons Australia, Ltd. The retraction has been agreed upon following an investigation into concerns raised by a third party which revealed inappropriate image panel duplications between this (Figure 1a and b) and three other articles from the same institute published in a different scientific context. The authors admitted to the image compilation error and stated that during the experimental work, the author groups shared a laboratory which could have led to the error in reusing the images. Due to the number of errors identified in the published figures, the authors and the editors have lost confidence in the presented data and consider the conclusions substantially compromised.

